# A GENOME-WIDE CRISPR/CAS9 SCREEN REVEALS EPIGENETIC IMMUNE EVASION MECHANISMS IN HUMAN PAPILLOMAVIRUS-POSITIVE HEAD AND NECK SQUAMOUS CELL CARCINOMA

**DOI:** 10.51894/001c.122809

**Published:** 2024-08-30

**Authors:** Nicholas S. Giacobbi, Lexi Vu, Shreya Mullapudi, Canchai Yang, Niklas A. Wahl, Mohamed Khalil, Andrew Olive, Dohun Pyeon

**Affiliations:** 1 College of Osteopathic Medicine Michigan State University

## 7

### INTRODUCTION

Human papillomavirus-positive head and neck squamous cell carcinoma (HPV+ HNSCC) has limited expression of MHC-I molecules for antigen presentation, resulting in immune evasion and limited efficacy of immune checkpoint inhibitor (ICI) therapy.

### OBJECTIVE

In HPV+ HNSCC, we sought to identify negative regulators of MHC-I expression and to elucidate the underlying mechanism(s). Identifying new targets to increase MHC-I antigen presentation may improve ICI efficacy.

### METHODS:/ HYPOTHESIS

We performed genome-wide CRISPR/Cas9 screens in HPV+ HNSCC cell lines and identified the components of the polycomb repressive complex 2 (PRC2) as significant negative regulators of MHC-I. PRC2 represses MHC-I-related gene expression via histone methylation, and expression of the catalytic core protein of PRC2, EZH2, is upregulated in HPV+ cancers. Thus, we hypothesize that PRC2-mediated transcriptional repression downregulates MHC-I expression in HPV+ HNSCC.

### RESULTS

Individual knockouts of key PRC2 genes, EZH2, EED, and SUZ12, identified in our CRISPR/Cas9 screens as negative regulators of MHC-I, showed restored MHC-I expression in HPV+ HNSCC cells. Ingenuity Upstream Regulator Analysis predicted the transcription factor E2F1 as the most significant upstream regulator of the genes for MHC-I downregulation. Overexpression of E2F family members upregulated EZH2 and downregulated MHC-I expression in HPV+ HNSCC cells. Additionally, an epigenetic regulator complex, Spt-Ada-Gcn5 acetyltransferase (SAGA), was significantly enriched among negative regulators. The SAGA complex regulates gene expression via histone acetyltransferase activity through the lysine acetyltransferase 2A (KAT2A/GCN5). We show that KAT2A is upregulated in HPV+ HNSCC cells, KAT2A knockout upregulated MHC-I expression, and that KAT2A directly binds to E2F1 protein. Lastly, treatment with EZH2 inhibitor (GSK126) or histone acetyltransferase inhibitor (Garcinol) increases MHC-I expression in HPV+ HNSCC cells, suggesting that KAT2A and E2F1 upregulate the expression of PRC2 components.

### CONCLUSIONS

HPV is known to disrupt the repression of E2F transcription factors by degradation of pRB via the viral oncogene E7. Further dysregulation of E2F signaling mediated via KAT2A may upregulate PRC2, subsequently downregulating MHC-I expression. Loss of MHC-I may contribute to tumor immune evasion and ICI therapy failure. However, targeting EZH2 or KAT2A may significantly increase MHC-I expression, and our work may provide novel targets that improve patient immunotherapy response.

